# Chitosan Films Functionalized with Different Hydroxycinnamic Acids: Preparation, Characterization and Application for Pork Preservation

**DOI:** 10.3390/foods10030536

**Published:** 2021-03-05

**Authors:** Huimin Yong, Yunpeng Liu, Dawei Yun, Shuai Zong, Changhai Jin, Jun Liu

**Affiliations:** College of Food Science and Engineering, Yangzhou University, Yangzhou 225127, China; yhm2021@126.com (H.Y.); lyp1262021@126.com (Y.L.); daweiyun2021@126.com (D.Y.); shuaizong@yzu.edu.cn (S.Z.); chjin@yzu.edu.cn (C.J.)

**Keywords:** chitosan, hydroxycinnamic acid, graft copolymer, active packaging films, pork storage

## Abstract

Hydroxycinnamic acids are one category of bioactive phenolic acids that are widely distributed in plants. In this study, chitosan (CS) was functionalized with three kinds of hydroxycinnamic acids (*p*-coumaric acid, caffeic acid and ferulic acid) through the carbodiimide-mediated grafting method. The obtained hydroxycinnamic-acid-grafted CSs (hydroxycinnamic acid-g-CSs) were further fabricated into food packaging films through solvent casting. For the first time, the functionalities of the different hydroxycinnamic acid-g-CS films were compared. Results showed the grafting ratio of *p*-coumaric acid-g-CS, caffeic acid-g-CS and ferulic acid-g-CS was 73.68, 129.42 and 91.75 mg/g, respectively. Instrumental analyses confirmed hydroxycinnamic acids conjugated with CS through amide and ester bonds. The functionalization of CS film with hydroxycinnamic acids produced a more compact microstructure and higher UV light barrier ability, mechanical strength, water vapor barrier ability, thermal stability and antioxidant and antimicrobial activities. Among the different hydroxycinnamic acid-g-CS films, caffeic acid-g-CS film presented the strongest barrier, mechanical, antioxidant and antimicrobial properties. Moreover, caffeic acid-g-CS film packaging effectively extended the shelf life of pork to 10 days at 4 °C. Our results suggest caffeic acid-g-CS film can be used in the active food packaging field.

## 1. Introduction

Nowadays, the development of active packaging films with multi-functionalities (e.g., carbon dioxide and ethylene scavenging, moisture absorbing and antioxidant and antimicrobial abilities) has received growing interest in the food industry [1]. Antioxidant and antimicrobial packaging films have been widely studied in order to inhibit oxidation and pathogen growth in food [2,3,4]. Chitosan (CS), a positive-charged polysaccharide, is often selected as the matrix of active packaging films due to its excellent renewable, antimicrobial and film-forming properties [5]. Meanwhile, the abundant amino and hydroxyl groups amongst CS molecules provide opportunities to improve the functionality of CS films by structural modification [6,7].

Phenolic compounds, mainly classified as phenolic acids, flavonoids, tannins, stilbenes and lignans, are the secondary metabolites of vascular plants [8]. The antioxidant and antimicrobial activities of phenolic compounds have been widely demonstrated by many researchers [9,10]. Therefore, phenolic compounds are normally directly incorporated into the CS matrix to fabricate active packaging films [11]. However, the direct incorporation of phenolic compounds into CS films has some disadvantages, such as the low stability and rapid release of active substances [12]. Recent studies have shown phenolic compounds can be covalently conjugated onto CS molecules by graft copolymerization reactions [7]. The obtained phenolic-grafted-CSs (phenolic-g-CSs) not only present higher stability than free phenolic compounds but also possess stronger antioxidant and antimicrobial activities than unmodified CS [13]. In this respect, several active packaging films have been developed by using phenolic-g-CSs [14,15,16,17]. The developed phenolic-g-CS films are more stable than the CS/phenolic blend films. In addition, the grafted phenolic moieties are hardly released from the film matrix and thus phenolic-g-CS films exhibit a long-term antioxidant and antimicrobial efficiency [18,19]. As reported, the functionality of phenolic-g-CS films is influenced by the grafting ratio [20], the grafting method [17] and the type of grafted phenolic compounds [15,16].

Hydroxycinnamic acids are one category of phenolic acids that consist of a phenylpropanoid C6–C3 structure [21]. *p*-Coumaric acid, caffeic acid and ferulic acid are the most commonly studied hydroxycinnamic acids that differ in the position and number of hydroxyl and methoxyl groups. As reported, the antioxidant and antimicrobial activities of the hydroxycinnamic acids are closely associated with the arrangement of the hydroxyl and methoxyl groups [22]. Till now, only few studies have focused on the fabrication of active packaging films by using hydroxycinnamic acid-g-CSs (e.g., caffeic acid-g-CS and ferulic acid-g-CS) [14,16,23]. Moreover, the functionality of the different hydroxycinnamic acid-g-CS films has never been compared.

Fresh pork is one of the most consumed meats in the world. However, fresh pork is highly perishable because its abundant polyunsaturated fatty acids and proteins are sensitive to microbial spoilage and oxidation, which shortens the shelf life of pork and leads to economic loss [24]. Till now, active packaging has been demonstrated as an effective way to extend the shelf life of pork [25]. CS films incorporated with different kinds of antimicrobial and antioxidant agents (e.g., tea polyphenols, essential oil, green tea and spice extracts) have been developed and applied for pork preservation [26,27,28,29]. Nonetheless, no study has been conducted to maintain the quality and extend the shelf life of pork by phenolic-g-CS films.

In this study, CS was functionalized with three kinds of hydroxycinnamic acids (*p*-coumaric acid, caffeic acid and ferulic acid) through the carbodiimide-mediated grafting method. The synthesized hydroxycinnamic acid-g-CSs were characterized and then fabricated into packaging films by solvent casting. The physical and functional properties of the different hydroxycinnamic acid-g-CS films were compared. Finally, the hydroxycinnamic acid-g-CS film with the best functionality was used for pork packaging.

## 2. Materials and Methods

### 2.1. Materials and Reagents

Chitosan (CS) (deacetylated degree: 90%; average molecular weight: 1.5 × 10^5^ Da), *p*-coumaric acid, caffeic acid, ferulic acid, 1-ethyl-3-(3-dimethylaminopropyl) carbodiimide hydrochloride (EDC), N-hydroxysuccinimide (NHS) and 2,2-diphenyl-1-picrylhydrazyl (DPPH) were purchased from Macklin Biochemical Co., Ltd. (Shanghai, China). Fresh pork was purchased from a local market (Yangzhou, China).

### 2.2. Synthesis of Hydroxycinnamic Acid-g-CSs

Hydroxycinnamic acid-g-CSs were obtained by carbodiimide-mediated coupling [15]. Briefly, each hydroxycinnamic acid (3.75 mmol) was reacted with EDC (3.75 mmol) and NHS (3.75 mmol) in 30 mL of 2-(*N*-morpholino)ethanesulfonic acid buffer (pH 5.5) at 20 °C for 1 h. Then, the reaction solution was mixed with 25 mL of 7.5 mmol CS and further reacted at 20 °C for 24 h. The product was dialyzed against distilled water for 24 h and centrifuged at 10,000× *g* for 40 min to remove the unconjugated hydroxycinnamic acid. The obtained supernatant was further dialyzed against distilled water for 48 h and freeze-dried. TLC analysis was performed to check whether the dialyzate contained free hydroxycinnamic acid [30]. The grafting ratio of the hydroxycinnamic acid-g-CSs was measured by Folin–Ciocalteu assay [17].

### 2.3. Characterization of Hydroxycinnamic Acid-g-CSs

The successful conjugation of hydroxycinnamic acids onto CS was verified by UV-vis spectroscopy, Fourier-transform infrared (FT-IR) spectroscopy and X-ray diffraction (XRD) according to the method of Liu et al. [15].

### 2.4. Preparation of Hydroxycinnamic Acid-g-CS Films

Hydroxycinnamic acid-g-CS (3.2 g) and glycerol (0.96 g) were thoroughly mixed in 160 mL of an acetic acid solution (1%, *v*/*v*). The obtained solutions were degassed and poured into plates (24 cm × 24 cm) and dried at 30 °C for 2 days. The dried films were stored in a desiccator at 20 °C with 50% relative humidity until equilibrium [15].

### 2.5. Characterization of Hydroxycinnamic Acid-g-CS Films

The physicochemical properties of the films were determined by our previously established methods [31]. Film color parameters, including L*, a*, b* and total color difference (ΔE), were recorded by a SC-80C colorimeter (Kangguang Instrument Co., Beijing, China). Light transmittance of the films was measured on a Lambda 35 spectrophotometer (PerkinElmer Ltd., Waltham, MA, USA) at 200–800 nm. The water vapor permeability (WVP) of the films was tested by the gravimetric method according to ASTM E96-00. Tensile strength (TS) and elongation at break (EAB) of the films were determined by a TMS-Pro texture analyzer (Food Technology Co., Sterling, VA, USA). Thermal stability of the films was measured on a Pyris 1 thermogravimetric analysis (TGA) instrument (PerkinElmer Ltd., Waltham, MA, USA) at 30–800 °C. The film cross-section was observed using a Gemini 300 scanning electron microscope (Carl Zeiss, Oberkochen, Baden-Württemberg, Germany) at 5 kV and 600× magnification.

### 2.6. Antioxidant and Antimicrobial Activities of Hydroxycinnamic Acid-g-CS Films

The antioxidant activity of the films was tested by DPPH scavenging assay [31]. The antimicrobial activity of the films against four foodborne pathogenic bacteria strains, including *Escherichia coli* ATCC 43895, *Salmonella typhimurium* ATCC 14028, *Staphylococcus aureus* ATCC 6538 and *Listeria monocytogenes* ATCC 19115, was tested by the agar plate diffusion method [32].

### 2.7. Application of the Film in Pork Preservation

After removing visible connective tissue and fat, fresh pork was cut into small samples (approximately 100 g). Then, the pork samples were randomly assigned to three treatment groups: control group (samples unwrapped with film), CS film treatment group (samples wrapped with CS film) and caffeic acid-g-CS film treatment group (samples wrapped with caffeic acid-g-CS film). Then, all pork samples were stored in sterile plastic boxes at 4 °C for 14 days [33]. The pork samples were randomly taken out from each treatment group for characterization every 2 days.

#### 2.7.1. Determination of Total Volatile Basic Nitrogen (TVBN) Value

To evaluate the TVBN value of pork, the pork sample (10 g) was homogenized with 50 mL distilled water. After the solution was filtrated, the filtrate (5 mL) was mixed with 5 mL of MgO solution (10 g/L). Steam distillation was carried out by the Kjeldahl distillation Unit (Jinan Hanon Instruments Co., Ltd., Jinan, China) for 5 min. The distillate was collected in a receiving flask containing 10 mL of boric acid (20 g/L) and 5 drops of methyl red (0.1%) and bromocresol green (0.1%). Subsequently, the obtained solution was titrated with HCl (0.01 mol/L). The TVBN value was determined and expressed as mg/100 g pork based on the consumption of HCl [34].

#### 2.7.2. Determination of pH Value

To evaluate the pH value of pork, the pork sample (10 g) was homogenized with 100 mL distilled water at 8000 rpm for 5 min. Then, the pH value of the sample solution was determined using a pH meter (Kangyi Instrument Co., Ltd., Shanghai, China) [35].

#### 2.7.3. Determination of Total Viable Counts (TVC)

To evaluate the TVC of pork, the minced pork sample (25 g) was thoroughly mixed and homogenized with 225 mL of sterile physiological saline (0.9%) in a sterile airtight bag for 20 min, which was followed by a 10 times serial decimal dilution. Then, each diluted solution (0.1 mL) was spread on a nutrient broth agar plate and incubated at 37 °C for 48 h. The TVC was counted and expressed as log CFU/g [36].

#### 2.7.4. Determination of Thiobarbituric Acid Reactive Substances (TBARS) Value

To evaluate the TBARS value of pork, the pork sample (5 g) was thoroughly homogenized with 50 mL of trichloroacetic acid (7.5%) and ethylenediaminetetraacetic acid (0.1%) with shaking at 50 °C for 30 min. Then, the mixture was centrifuged and filtered. Afterwards, the filtrate (5 mL) was reacted with thiobarbituric acid (5 mL, 0.02 mol/L) at 90 °C for 30 min. After being cooled to room temperature, the absorbance of the reaction solution was measured at 532 nm. The TBARS value was calculated according to a standard curve of 1,1,3,3-tetramethoxypropane and expressed as mg malonaldehyde (MDA)/kg pork [34].

#### 2.7.5. Sensory Evaluation

The sensory attributes of the pork, including the color, odor and over acceptance, were determined by the modified method of Zhao et al. [37]. The sensory evaluation was performed by ten trained members from the College of Food Science and Engineering, Yangzhou University, in separate booths under a controlled environment. Different pork samples were individually presented to each panelist. Meanwhile, a fresh pork sample was offered to panelists for comparison with the treatment samples. Sensory attributes were scored by the ten panelists using a 10-point scale (10–9 = excellent, 8–7 = good, 6–5 = unacceptable, 4–3 = poor and 2–1 = very poor).

### 2.8. Statistical Analysis

Results were analyzed by SPSS 13.0 software (SPSS, Inc., Chicago, IL, USA) using Duncan’s test and one-way analysis of variance. Results were considered statistically different if *p* < 0.05.

## 3. Results

### 3.1. Synthesis of Hydroxycinnamic Acid-g-CSs

Till now, hydroxycinnamic acid-g-CSs have been synthesized by different grafting methods, such as enzyme catalysis, free radical-induced grafting and carbodiimide-mediated coupling methods [16,38,39,40,41]. Our previous study has shown carbodiimide-mediated coupling is the most effective method to synthesize phenolic acid-g-CSs [17]. In this study, three kinds of hydroxycinnamic acid-g-CSs were synthesized by the carbodiimide-mediated coupling reaction. As exhibited in Figure S1, the carboxyl groups on the hydroxycinnamic acids first react with EDC to form O-acylisourea, which subsequently undergoes a reaction with NHS to produce NHS ester. CS finally reacts with the NHS ester to yield hydroxycinnamic acid-g-CS [7].

TLC was applied to confirm the absence of free hydroxycinnamic acids in the hydroxycinnamic acid-g-CSs. As shown in Figure S2, free hydroxycinnamic acids migrated long distances and presented yellow spots on the plate after being exposed to iodine vapors and UV light. This was because iodine vapor could be adsorbed onto hydroxycinnamic acids [42]. However, three hydroxycinnamic acid-g-CSs only presented yellow spots at the baseline of the plate without any substance migration. Similarly, Liu et al. [30] and Chatterjee et al. [38] also confirmed hydroxycinnamic acid-g-CSs did not contain un-grafted hydroxycinnamic acids, by TLC test. The presence of phenolic groups in the hydroxycinnamic acid-g-CSs was checked by a Folin–Ciocalteu assay. The grafting ratio of *p*-coumaric acid-g-CS, caffeic acid-g-CS and ferulic acid-g-CS was 73.68, 129.42 and 91.75 mg/g, respectively. Among the three hydroxycinnamic acid-g-CSs, caffeic acid-g-CS presented the highest grafting ratio. Structurally speaking, three hydroxycinnamic acids had different substituents at C3 of the aromatic ring (Figure S1). Our results suggested that the substituent group at the C3-position greatly impacted the grafting efficiency of the hydroxycinnamic acid-g-CSs.

### 3.2. Characterization of the Hydroxycinnamic Acid-g-CSs

**Figure 1:**
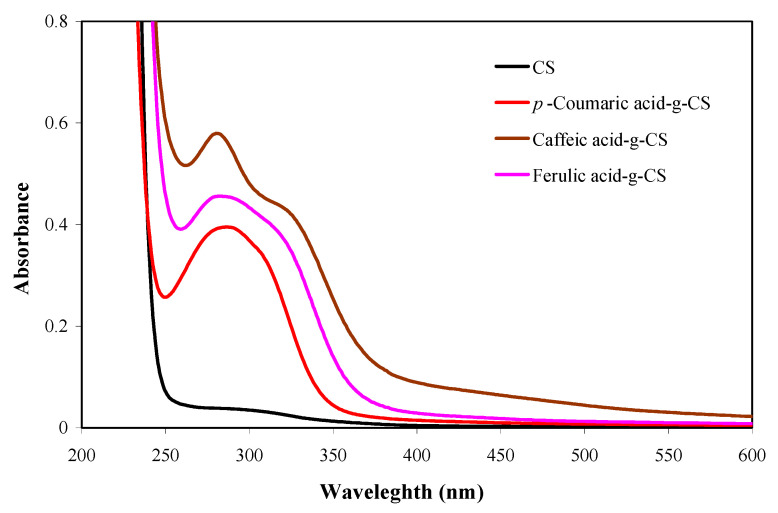
UV-vis spectra of chitosan (CS) and different hydroxycinnamic acid-g-CSs.

The successful conjugation of hydroxycinnamic acids with CS was verified through UV-vis spectroscopy, FT-IR spectroscopy and XRD. Different from CS, hydroxycinnamic acid-g-CSs displayed obvious UV-vis absorption peaks in the range of 280–330 nm (Figure 1), corresponding to the phenylpropanoid moieties in the hydroxycinnamic acids [43]. UV-vis spectra of the hydroxycinnamic acid-g-CSs were similar with previous reports [40,44,45,46,47]. However, hydroxycinnamic acid-g-CSs synthesized by enzyme catalysis presented different UV-vis spectra with absorption peaks appearing at about 300 and 350 nm, which was because the hydroxycinnamic acids were oxidized by enzymes [48]. In this study, the absorption peak intensity of the hydroxycinnamic acid-g-CSs was positively associated with their grafting ratios. Liu et al. [15] also observed a similar tendency in the UV-vis spectra of hydroxybenzoic acid-g-CSs.

**Figure 2:**
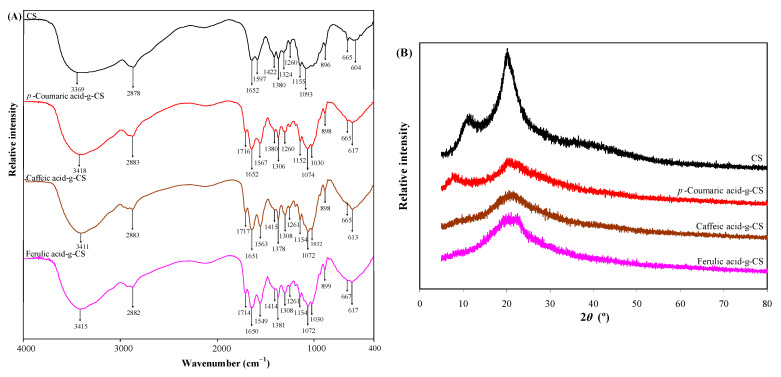
FT-IR spectra (**A**) and XRD patterns (**B**) of chitosan (CS) and different hydroxycinnamic acid-g-CSs.

Hydroxycinnamic acid-g-CSs presented different FT-IR spectra from CS (Figure 2A). The N−H and O−H stretching of CS shifted to 3411–3418 cm^−1^ after grafting with different hydroxycinnamic acids, which was because the hydrogen bond interactions in the CS network were disrupted by the grafted hydroxycinnamic acids [40]. Meanwhile, hydroxycinnamic acid-g-CSs showed two new bands at 1714–1716 cm^−1^ and 1549–1567 cm^−1^, which corresponded to the C=O stretching of the ester bond and amide bond, respectively [15]. Our results implied hydroxycinnamic acids were grafted onto CS via ester and amide linkages. Other researchers found hydroxycinnamic acid-g-CSs prepared by carbodiimide-mediated coupling had similar FT-IR spectra [38]. However, some studies reported hydroxycinnamic acid-g-CSs obtained by enzymatic catalysis had different FT-IR spectra, which was because the Schiff base (C=N stretching) and Michael type adduct (C−N stretching) were produced in the grafting process [17,41]. Therefore, the FT-IR spectra of the hydroxycinnamic acid-g-CSs were related to the grafting method.

The crystalline character of the hydroxycinnamic acid-g-CSs was evaluated by XRD (Figure 2B). The semi-crystalline structure of CS was somewhat destroyed after grafting with hydroxycinnamic acids. For CS, its diffraction peaks at 11° and 20° significantly decreased. The crystalline character changes were caused by the weakening of hydrogen bond interactions within CS after grafting with hydroxycinnamic acids. Other researchers also observed the graft of hydroxycinnamic acids decreased the crystalline degree of CS [46,47,49]. Notably, three hydroxycinnamic acid-g-CSs showed different XRD patterns. Caffeic acid-g-CS exhibited the lowest crystalline degree because it had the highest grafting ratio.

### 3.3. Characterization of Hydroxycinnamic Acid-g-CS Films

#### 3.3.1. Color

**Figure 3:**
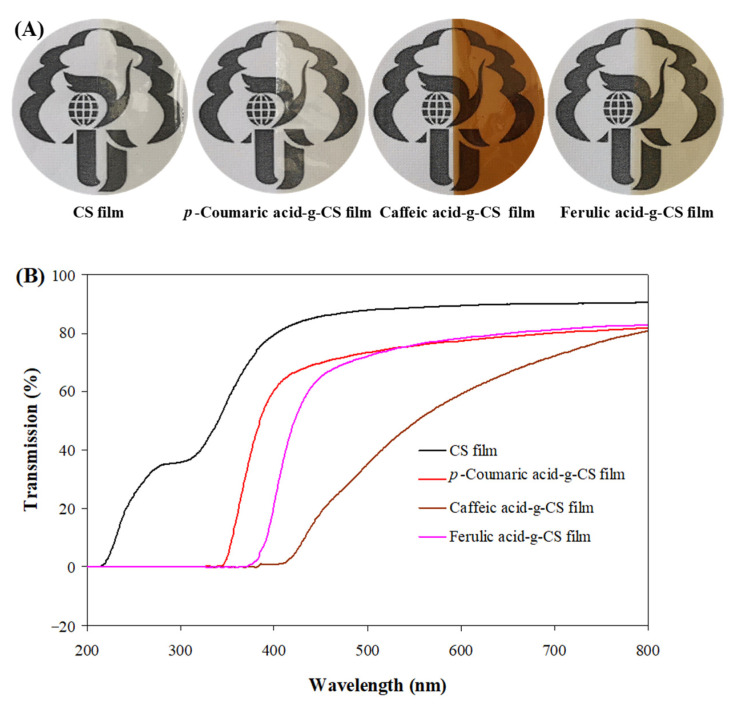
Physical appearances (**A**) and UV-vis light transmittance (**B**) of chitosan (CS) and different hydroxycinnamic acid-g-CS films.

As presented in Figure 3A, the color of the hydroxycinnamic acid-g-CS films was related to the type of grafted hydroxycinnamic acids. Hydroxycinnamic acid-g-CS films had similar colors to the un-grafted hydroxycinnamic acids. Such similar results also were reported by Liu et al. [15] in different hydroxybenzoic acid-g-CS films. The appearances of the hydroxycinnamic acid-g-CS films were consistent with their color parameters (Table 1). The graft of hydroxycinnamic acids made the films became dark (L* value decreased) and yellow (b* value increased). Among the different films, caffeic acid-g-CS film had the highest a*, b* and ΔE values because caffeic acid-g-CS had the highest grafting ratio (*p* < 0.05).

#### 3.3.2. Light Transmittance

UV-vis light can cause the quality loss of food and thus it is important for packaging films to have the desired light barrier potential. The graft of hydroxycinnamic acids remarkably decreased the film light transmittance (Figure 3B), which was caused by the absorption ability of C=O and C=C in the grafted hydroxycinnamic acids [50]. Many other researchers also demonstrated the conjugation of hydroxybenzoic acids reduced the light transmittance of CS films [15,17,20]. Although three hydroxycinnamic acid-g-CS films possessed similar chromophore structures, the light transmittance of these films was somewhat different. This was mainly because the three hydroxycinnamic acid-g-CSs had different grafting ratios. In this respect, caffeic acid-g-CS possessed the lowest light transmittance. Liu et al. [20] also found the light transmittance of the protocatechuic acid-g-CS films decreased with the elevation of the grafting ratio.

#### 3.3.3. WVP

The packaging films provide a water vapor barrier between the food and external environment, which is normally indicated by WVP. The conjugation of the hydroxycinnamic acids lowered the WVP of the CS films (Table 2). Other researchers also observed the WVP of the CS films decreased after grafting with ferulic acid and caffeic acid [14,16]. This decrease in the WVP of the hydroxycinnamic acid-g-CS films could have several reasons. Firstly, the covalent bonds (ester and amide linkages) between the hydroxycinnamic acid and CS made the number of hydrophilic hydroxyl and amino groups amongst the CS decrease [14]. Secondly, the grafted hydroxycinnamic acids were more hydrophobic than CS, resulting in a decreased water vapor affinity of the films [49]. Thirdly, the grafted hydroxycinnamic acids could fill in the gaps between the CS chains, causing the formation of a denser structure of the films [15]. Due to its highest grafting ratio, caffeic acid-g-CS film presented the lowest WVP (*p* < 0.05). Liu et al. [15] also observed the WVP of hydroxybenzoic acid-g-CS films decreased with the elevation of the grafting ratio.

#### 3.3.4. Mechanical Property

Food packaging films should possess proper TS and EAB to withstand external mechanical force and deformation. Hydroxycinnamic acid-g-CS films showed significantly higher TS and EAB than CS film (Table 2), suggesting the mechanical property of the films was elevated after grafting with hydroxycinnamic acids. The elevated TS was attributed to the establishment of a more compact network in hydroxycinnamic acid-g-CS films through intermolecular hydrogen bonds [12,16]. Meanwhile, the formed continuous and cohesive film matrix could increase the flexibility of the hydroxybenzoic acid-g-CS films, thereby increasing the EAB of the films [15]. Similarly, Liu et al. [15] observed the grafting of different hydroxybenzoic acids enhanced the TS and EAB of CS films. In addition, they found the mechanical property of the films was influenced by the type of grafted hydroxybenzoic acids [15]. Notably, caffeic acid-g-CS film presented the highest TS and EAB due to its highest grafting ratio (*p* < 0.05). Some researchers also documented the grafting ratio of hydroxybenzoic acid-g-CS films greatly affected their mechanical property [12,15,20].

#### 3.3.5. Thermal Property

**Figure 4:**
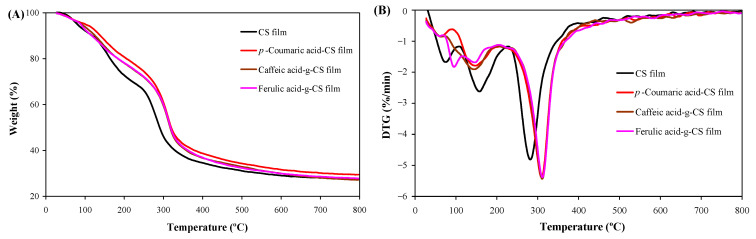
Thermogravimetric analysis (TGA) (**A**) and derivative TGA (DTG) (**B**) profiles of chitosan (CS) and different hydroxycinnamic acid-g-CS films.

The TGA and derivative TGA (DTG) curves showed the weight loss of the films could be divided into three phases (Figure 4). The first phase (30–126 °C) was caused by the evaporation of acetic acid and moisture in the films [20]. The second and the third phases (127–226 °C and 227–800 °C) were associated with the decomposition of glycerol and hydroxycinnamic acid-g-CSs, respectively [17]. Notably, hydroxycinnamic acid-g-CS films showed relatively slower degradation rates than CS film. Meanwhile, the maximum decomposition rate of the hydroxycinnamic acid-g-CS and CS films appeared at about 313 and 285 °C, respectively. These results indicated hydroxycinnamic acid-g-CS films had higher thermal stability, which was because the CS and hydroxycinnamic acids had formed stable ester and amide linkages. However, different hydroxycinnamic acid-g-CS films did not have remarkable differences in thermal stability. Our results suggested the thermal stability of the hydroxycinnamic acid-g-CS films was not remarkably affected by the kind of grafted hydroxycinnamic acids.

#### 3.3.6. Microstructure

**Figure 5:**
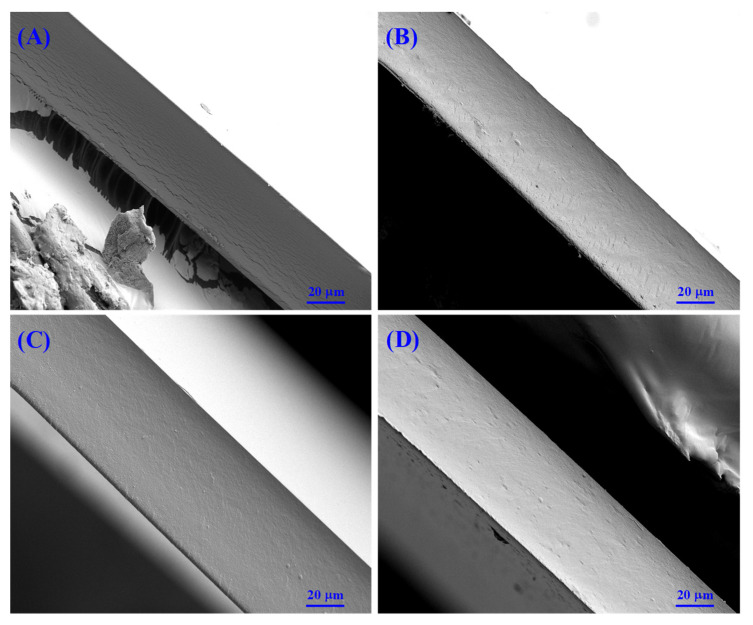
SEM micrographs on the cross-section of chitosan (CS) (**A**), *p*-coumaric acid-g-CS (**B**), caffeic acid-g-CS (**C**) and ferulic acid-g-CS (**D**) films.

SEM observation can contribute to the understanding of film microstructure. As shown in Figure 5, the CS film had a fractured cross-section, which could be associated with the semi-crystalline character of CS molecules [51]. By contrast, hydroxycinnamic acid-g-CS films had tight cross-sections without cracks. Similarly, some researchers also observed the cross-section of the protocatechuic acid-g-CS and caffeic acid-g-CS films was relatively tighter than CS film, which was because these phenolic acid-g-CSs were amorphous and more compatible with the plasticizer of glycerol [16,20]. In addition, the hydroxyl groups in the grafted hydroxycinnamic acids could interact with CS backbones and glycerol through inter-molecular hydrogen bonds [12]. Since caffeic acid had two hydroxyl groups, it had more opportunities to interact with CS backbones and glycerol. Meanwhile, the relatively higher grafting ratio of caffeic acid-g-CS further provided more hydroxyl groups. As a result, the hydrogen bond interactions within caffeic acid-g-CS film were stronger than other hydroxycinnamic acid-g-CS films. Therefore, caffeic acid-g-CS film presented the lowest WVP and highest TS and EAB among all the films.

#### 3.3.7. Antioxidant Activity

**Figure 6:**
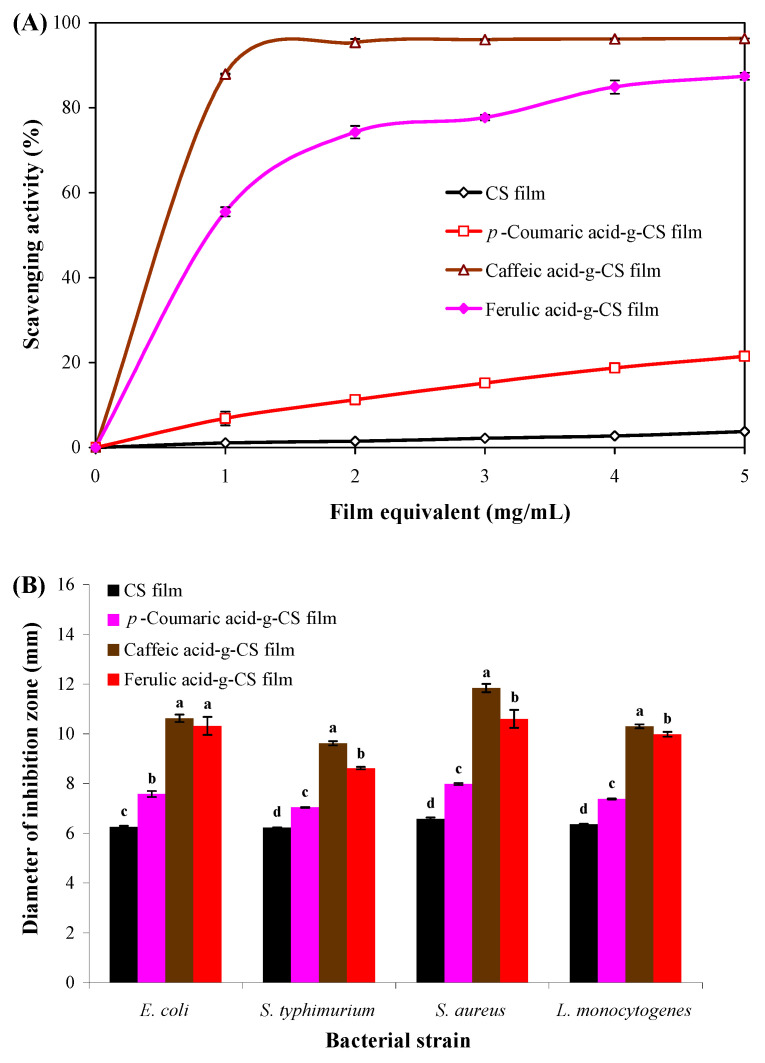
DPPH radical scavenging activity (**A**) and antimicrobial activity (**B**) of chitosan (CS) and different hydroxycinnamic acid-g-CS films. Data are presented as the mean ± SD of triplicates. Different lowercase letters indicate a statistically significant difference among treatments against the same bacterial strain (*p* < 0.05).

Hydroxycinnamic acid-g-CS films presented stronger antioxidant activity than CS film (*p* < 0.05) (Figure 6A), which was because the grafted hydroxycinnamic acids had potent hydrogen donating ability [14]. Several previous studies also reported the functionalization of CS films with hydroxycinnamic acids could enhance the antioxidant activity of the films [14,16]. In this study, the antioxidant activity of hydroxycinnamic acid-g-CS films was related to the kind of grafted hydroxycinnamic acids. Caffeic acid-g-CS film had the strongest antioxidant activity (*p* < 0.05) mainly because it had the highest grafting ratio and provided abundant phenolic hydroxyl groups to scavenge free radicals. In addition, caffeic acid had two hydroxyl groups in its structure, which further contributed to the higher antioxidant activity of caffeic acid-g-CS film [52]. Liu et al. [15] also reported the antioxidant activity of hydroxybenzoic acid-g-CS films was affected by the kind of grafted hydroxybenzoic acids and the grafting ratio. Recently, Zhang et al. [17] found gallic acid-g-CS film prepared by carbodiimide-mediated coupling had higher antioxidant activity than that prepared by free radical-induced grafting and enzyme catalysis, suggesting the antioxidant activity of the films was affected by the grafting method. Our results showed caffeic acid-g-CS film had good potential as an antioxidant packaging film.

#### 3.3.8. Antimicrobial Activity

The application potential of hydroxycinnamic acid-g-CS films as antimicrobial packaging was evaluated by an agar plate diffusion test (Figure 6B). The antimicrobial activity of CS film has been well demonstrated, which was due to the interactions between the CS and microbial cell membrane [53]. After grafting with hydroxycinnamic acids, the antimicrobial activity of the films was remarkably elevated. As reported, hydroxycinnamic acids can disrupt cell membranes and cause cytoplasmic leakage [22,54]. A recent study also documented the antimicrobial activity of CS film was improved after grafting with caffeic acid [16]. Since caffeic acid-g-CS film had the highest grafting ratio, it presented the strongest antimicrobial activity. This suggested caffeic acid-g-CS film could be further utilized in antimicrobial packaging.

### 3.4. Application of Caffeic Acid-g-CS Film in Pork Packaging

Food pathogens and lipid oxidation are the main causes of pork spoilage, which can result in discoloration, off-flavor and nutritional loss of pork [55,56]. Hence, it is essential to develop active packaging to prolong the shelf life of pork. Since caffeic acid-g-CS film had the highest barrier, mechanical, antioxidant and antimicrobial properties, it was further used to package fresh pork.

**Figure 7:**
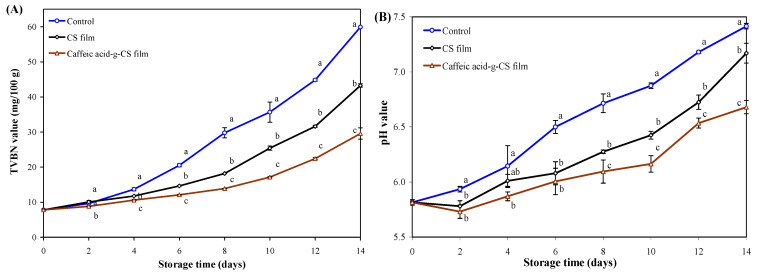

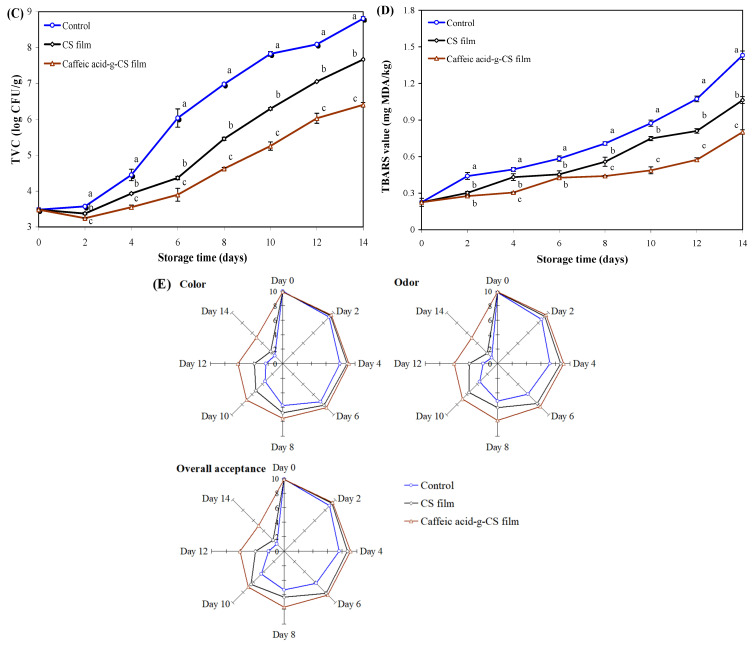
Changes in the total volatile basic nitrogen (TVBN) value (**A**), pH value (**B**), total viable counts (TVC) (**C**), Thiobarbituric acid reactive substances (TBARS) value (**D**) and sensory properties (**E**) of pork packaged in the control group, chitosan (CS) film and caffeic acid-g-CS film at 4 °C for 14 days. Data are presented as the mean ± SD of triplicates. Different lowercase letters indicate a statistically significant difference among treatments at the same storage time (*p* < 0.05).

TVBN, the main product of protein spoilage, is usually used to evaluate the degradation of proteins in pork [34]. As exhibited in Figure 7A, the TVBN value of pork gradually increased during storage. Notably, the TVBN value of pork packaged in the control group, CS film and caffeic acid-g-CS film exceeded the limitation of Chinese Standard GB 2707-2016 for fresh pork (15 mg/100 g) at the 6th, 8th and 10th day, respectively. Notably, the pH value of pork increased with the production of volatile nitrogen compounds (Figure 7B). By contrast, pork packaged by caffeic acid-g-CS film showed the lowest pH (*p* < 0.05). Meanwhile, the pork packaged by caffeic acid-g-CS film had a much lower TVC as compared to the control group and CS films (Figure 7C). The TVC of pork packaged in the control group, CS and caffeic acid-g-CS films exceeded the limitation of Chinese Standard GB/T 9959.2-2008 for fresh pork (6.00 log CFU/g) at the 6th, 10th and 12th day, respectively. The above results suggested caffeic acid-g-CS film could effectively prolong the shelf life of pork because the film had higher antimicrobial activity. Other researchers also documented CS films added with spice extract and tea polyphenols could inhibit an increase in TVBN, pH and TVC in pork during storage [26,27].

Lipid oxidation is one of the main causes of meat deterioration. The TBARS value is usually used to assess the lipid oxidation degree of pork. As exhibited in Figure 7D, the TBARS value of pork gradually increased during storage. Notably, pork packaged by caffeic acid-g-CS film exhibited the lowest TBARS value (*p* < 0.05), indicating the film could effectively retard lipid oxidation of pork. This was because the grafted caffeic acid moieties could capture active oxygen species and interrupt lipid oxidation. Similarly, some researchers also observed the incorporation of antioxidants (e.g., tea polyphenols, green tea extract and essential oil) into CS films significantly retarded the lipid oxidation in pork [27,28,29].

The sensory properties of pork, including color, odor and over acceptance, are shown in Figure 7E. Due to microbial spoilage and lipid oxidation in the pork, the sensory properties of pork packaged with all the films gradually decreased during storage. Since caffeic acid-g-CS film had the strongest antimicrobial and antioxidant activities, the pork packaged by the film presented the best sensory properties. Based on the criteria of sensory evaluation, the pork packaged in the control group, CS and caffeic acid-g-CS films became unacceptable at the 6th, 10th and 12th day, respectively. Based on the above results, caffeic acid-g-CS film could effectively extend the shelf life of pork to at least 10 days at 4 °C.

## 4. Conclusions

Hydroxycinnamic acids were covalently attached onto CS chains via amide and ester bonds. Among the different hydroxycinnamic acid-g-CSs, caffeic acid-g-CS exhibited the highest grafting ratio. The graft of hydroxycinnamic acids efficiently enhanced the compactness, UV light and water vapor barrier ability, mechanical property, thermal stability and antioxidant and antimicrobial activities of CS films. The functionality of hydroxycinnamic acid-g-CS films was affected by the kind of grafted hydroxycinnamic acids and the grafting ratio of the hydroxycinnamic acid-g-CSs. Since caffeic acid-g-CS film had the strongest water vapor barrier, antioxidant and antimicrobial properties, it extended the shelf life of pork to 10 days at 4 °C. In the future, the impact of caffeic acid-g-CS film packaging on other food products can be investigated.

## Figures and Tables

**Table 1:** Color parameters including L*, a*, b* and ΔE of chitosan (CS), *p*-coumaric acid-g-CS, caffeic acid-g-CS and ferulic acid-g-CS films.

Films	L*	a*	b*	ΔE
CS film	94.43 ± 0.02 ^a^	−1.16 ± 0.01 ^d^	2.12 ± 0.13 ^d^	3.42 ± 0.14 ^d^
*p*-Coumaric acid-g-CS film	92.58 ± 0.29 ^b^	−0.86 ± 0.04 ^b^	4.77 ± 0.71 ^c^	6.12 ± 0.74 ^c^
Caffeic acid-g-CS film	63.52 ± 0.19 ^d^	13.58 ± 0.13 ^a^	43.66 ± 0.20 ^a^	55.58 ± 0.29 ^a^
Ferulic acid-g-CS film	90.49 ± 0.08 ^c^	−1.00 ± 0.05 ^c^	11.30 ± 0.16 ^b^	12.90 ± 0.14 ^b^

Values are given as the mean ± SD (*n* = 3). Different lowercase letters in the same column indicate significant differences (*p* < 0.05).

**Table 2:** Water vapor permeability (WVP), tensile strength (TS) and elongation at break (EAB) of chitosan (CS), *p*-coumaric acid-g-CS, caffeic acid-g-CS and ferulic acid-g-CS films.

Films	WVP (10^−10^ g m^−1^ s^−1^ Pa^−1^)	TS (MPa)	EAB (%)
CS film	10.94 ± 0.22 ^a^	45.72 ± 3.42 ^d^	66.36 ± 2.14 ^d^
*p*-Coumaric acid-g-CS film	9.88 ± 0.06 ^b^	58.58 ± 5.97 ^c^	76.13 ± 5.91 ^c^
Caffeic acid-g-CS film	7.49 ± 0.37 ^d^	71.60 ± 2.61 ^a^	90.27 ± 2.15 ^a^
Ferulic acid-g-CS film	8.01 ± 0.16 ^c^	65.08 ± 3.25 ^b^	80.01 ± 7.36 ^b^

Values are given as the mean ± SD (*n* = 3 for WVP and *n* = 6 for TS and EAB). Different lowercase letters in the same column indicate significant differences (*p* < 0.05).

## Data Availability

The data presented in this study are available on request from the corresponding author.

## Supplementary Materials

The following are available online at https://www.mdpi.com/2304-8158/10/3/536/s1, Figure S1: Synthetic mechanisms of hydroxycinnamic acid-g-CSs by EDC/NHS mediated coupling, Figure S2: TLC of chitosan (CS) and different hydroxycinnamic acid-g-CSs.
