# Supplementary material for: Chitosan Films Functionalized with Different Hydroxycinnamic Acids: Preparation, Characterization and Application for Pork Preservation
Source: Foods. 2021 Mar 5;10(3):536. doi: 10.3390/foods10030536 (PMC8000987; doi:10.3390/foods10030536)
Supplement: Supplementary file 1 [file foods-10-00536-s001.pdf]

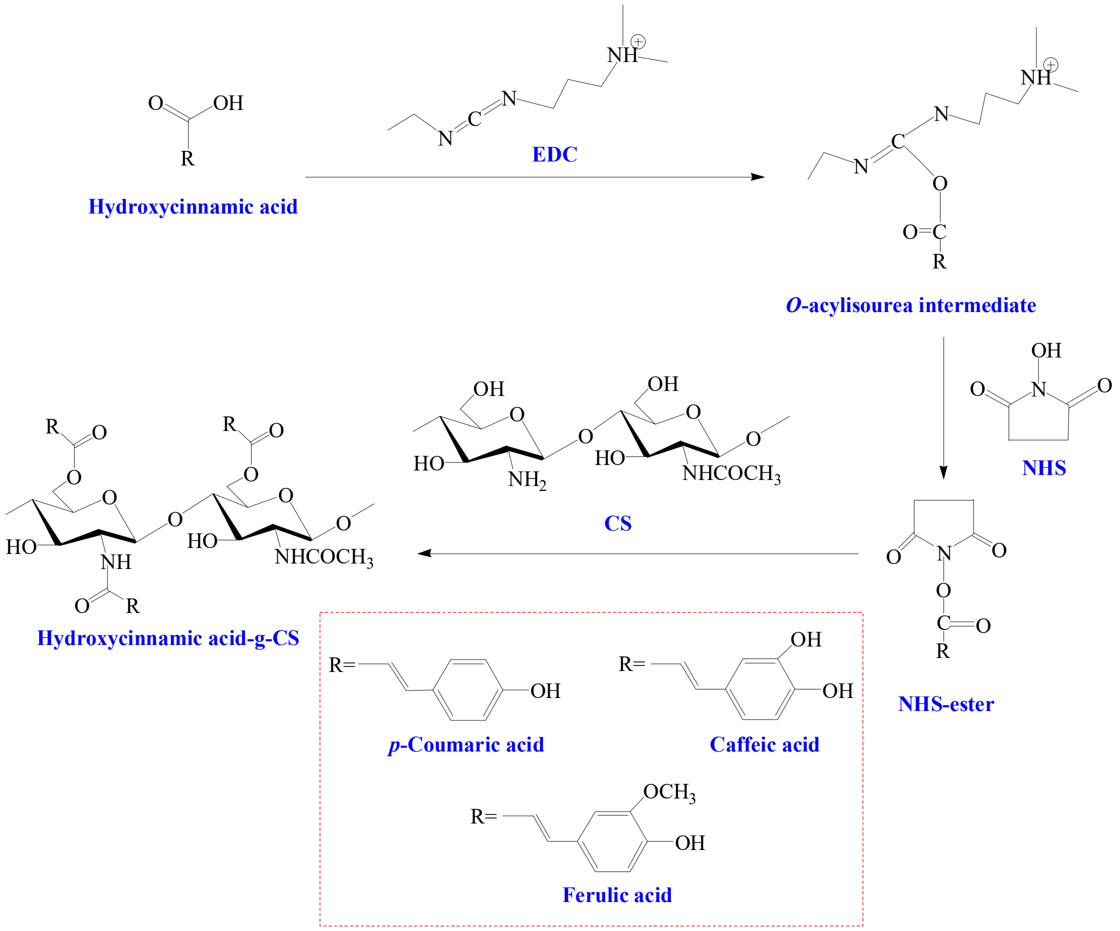


**Figure S1.** Synthetic mechanisms of hydroxycinnamic acid-g-CSs by EDC/NHS mediated coupling.

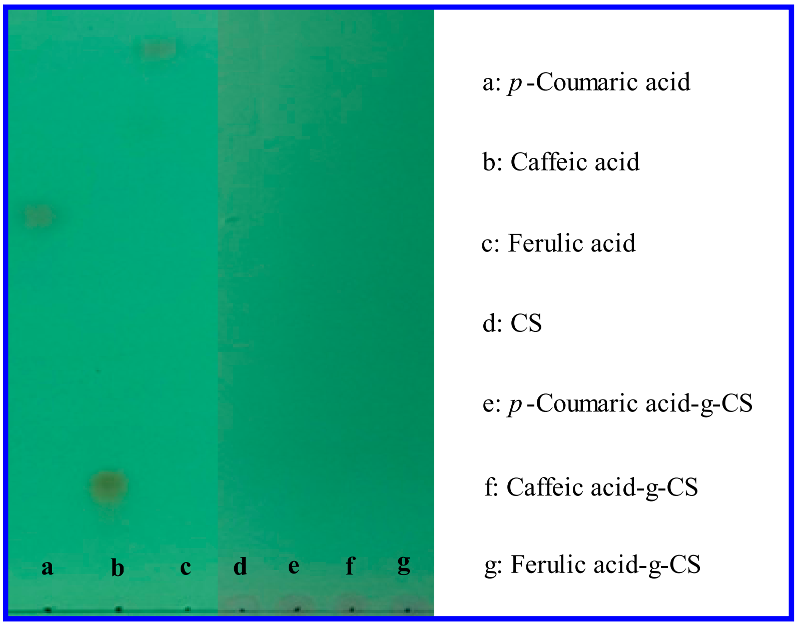


**Figure S2.** TLC of chitosan (CS) and different hydroxycinnamic acid-g-CSs.
